# The immune landscape and viral shedding of Omicron SARS-CoV-2 variants implicate immune escape

**DOI:** 10.3389/fmed.2024.1478466

**Published:** 2025-01-22

**Authors:** Weilong Zhang, Xiaoyan Gai, Zhonghui Duan, Changjian Yan, Chunyuan Huang, Chaoling Wu, Siping Zheng, Zixiang Lin, Qingtao Zhou, Lili Dai, Ping Yang, Fang Bao, Hongmei Jing, Chao Cai, Yingmin Ma, Yongchang Sun

**Affiliations:** ^1^Department of Hematology, Lymphoma Research Center, Peking University Third Hospital, Beijing, China; ^2^Department of Respiratory and Critical Care Medicine, Peking University Third Hospital, and Center for Chronic Airway Diseases, Peking University Health Science Center, Peking University, Beijing, China; ^3^Department of Respiratory and Critical Care Medicine, Beijing Youan Hospital, Capital Medical University, Beijing, China

**Keywords:** COVID-19, Omicron, viral shedding, immune escape, vaccination

## Abstract

**Background:**

Three years into the SARS-CoV-2 pandemic, the virus continues to mutate despite widespread vaccination, posing ongoing challenges for epidemic prevention and control. The relationship between viral shedding and immune escape remains under investigation. This study aims to examine the association between viral shedding and immune escape in the BA.4/5 and BF.7 variants.

**Method:**

We included 542 patients infected with the Omicron variant from Beijing Xiaotangshan shelter hospital. Based on the viral strain, patients were divided into BA.4/5 group and BF.7 group. Additionally, we categorized patients into rapid viral shedding and slow viral shedding groups according to their viral shedding rates. We explored the relationship between viral shedding and immune-related clinical indicators during this period.

**Result:**

Of the 542 patients, 118 were infected with BA.4/5 variant, and 424 were infected with BF.7 variant. The viral shedding duration differed significantly between BA.4/5 and BF.7 groups (*p* < 0.0001). However, there was no statistically significant correlation between viral shedding duration and immune-related indicators, such as WBC, Hb, PLT, Neu, Lym, CRP, allergy, fever, and vaccination status (*p* > 0.05). Furthermore, viral shedding duration was not associated with vaccination status, intervals between vaccinations, or vaccine types (*p* > 0.05).

**Conclusion:**

The duration of viral shedding in patients infected with Omicron variants BA.4/5 and BF.7 is not associated with WBC, Hb, Lym, CRP, fever, allergy, or vaccine-related indicators. This lack of association may be attributed to immune escape mechanisms.

## 1 Introduction

SARS-CoV-2 was first reported in Wuhan, China, in 2019 (1). The rapid extension of vaccination brought hope for controlling and preventing the spread of the virus. However, over the past 3 years, SARS-CoV-2 has continued to mutate. Omicron was first detected in Botswana on November 11th, 2021, and subsequently spread to many countries and regions (2). According to the Health Commission of the People’s Republic of China, as of December 31, 2022, 3.4 billion vaccine doses had been administered (3). Despite widespread vaccination, many individuals were still infected with Omicron. One study suggested that the protective effects of vaccines have diminished over time (4). Additionally, research of Ai et al. demonstrated that Omicron may have a greater ability to escape from vaccine-induced immune protection compared to other variants (5). Omicron is associated with a higher viral load and longer duration of viral shedding than Delta variant, especially in the nasopharynx (6). Peak viral shedding generally occurs 5–6 days after the onset of symptoms (7). BA.4/5 and BF.7 (also known as BA.5.2.1.7) subvariants of Omicron are highly contagious but have relatively low mortality rates (8), presenting new challenges for epidemic prevention and control. However, research on the relationship between viral shedding and immune escape for these two subvariants remains limited.

Viral shedding is primarily associated with viral load and is influenced by various factors, including gender (9, 10), age (11), and vaccination status (12). Xu et al. studied 113 symptomatic patients from two hospitals to identify risk factors related to viral shedding and found that prolonged SARS-CoV-2 RNA shedding was associated with gender; specifically, shedding duration was longer in males than in females (13). However, another study reported the opposite, suggesting that females had longer shedding durations (10). Moreover, other studies have identified age as an independent risk factor for those infected with the Omicron variant, with older individuals experiencing slower viral shedding (11). According to the research of Gao et al., prolonged viral shedding was strongly associated with increased C-reactive protein (CRP) and the decreased lymphocyte (Lym) counts (14). Additionally, it has been confirmed that lower lymphocyte and hemoglobin (Hb) levels, as well as higher neutrophil (Neu) counts, are independent risk factors for prolonged viral shedding (15). The severity of COVID-19 symptoms is also related to the duration of viral shedding. One study suggested that the viral shedding duration in asymptomatic COVID-19 patients was longer than in those with mild symptoms (16), while Kissler et al. presented a contrasting view (17). A study by Li et al. showed that fever was associated with prolonged viral shedding in individuals infected with COVID-19 (18).

A retrospective cohort study from China compared the effectiveness of two types of vaccines (BIBP and CoronaVac) against the Delta and Omicron variants (19). The study revealed that receiving two or three doses of the vaccines did not reduce the duration of viral shedding in patients infected with the Omicron variant, while vaccination was more effective in shortening viral shedding duration for the Delta variant. This suggested that the immune escape ability of Omicron was stronger than that of Delta. Concurrently, other studies showed that vaccination could shorten the duration of viral shedding in patients infected with Delta variant (12). Whereas, a study conducted at Boston University demonstrated that there was no difference in culture conversion time based on the variants or vaccination status (20).

The duration of virus shedding for the Omicron variant has shown highly variable across different studies. As the virus continues to mutate, the relationship between viral shedding and immune escape in patients infected with Omicron BA.4/5 and BF.7 variants remains uncertain. Based on the aforementioned studies, we performed the study in order to investigate the correlation between viral shedding and immune escape in the two variants.

## 2 Methods and materials

### 2.1 Participant recruitment and data collection

The study was conducted in Xiaotangshan Shelter Hospital of Beijing, China. Participants were recruited from May to June 2022 for those infected with Omicron BA.4/5, and from October to November 2022 for those infected with Omicron BF.7. The identification of Omicron BA.4/5 and BF.7 variants was aligned with reports of the Chinese CDC and GISAID during the two-epidemic period. A total of 542 hospitalized participants were enrolled in this study. The inclusion criteria were as follows: the nasal swab sample of each participant was tested at the testing center of the Beijing Xiaotangshan shelter hospital and the results of nucleic acid detection were positive. Asymptomatic patients were defined as those who tested positive nucleic acid detection but showed no clinical symptoms, while mild patients had mild symptoms but no evidence of pulmonary infection on CT scan. Both asymptomatic and mild patients were included in the study. The exclusion criteria included patients with no nucleic acid test reports or those in critical condition. Demographic data (age, sex), clinical characteristics (allergy, fever, diagnose, vaccine, WBC, Hb, PLT, Neu, Lym, CRP, types of vaccine and interval time between vaccinations) and virological data (duration of viral shedding) were collected form electronic medical record. Vaccine types were categorized into three main groups, including BIBP, CoronaVac and others. Participants were divided into BA.4/5 infection group and BF.7 infection group (21). The study adhered to the guidelines of the Declaration of Helsinki and was supported by the Ethics Committee of Peking University Third Hospital.

### 2.2 Examination of viral shedding by qPCR

Nasal swab detection reagents, targeting the Nucleocapsid (N) and open reading frame lab (ORF lab), were supplied by Shanghai Biogerm Medical Technology Co., Ltd. Each patient was tested an average of seven times. The viral load was measured by qPCR (22) and expressed as cycle threshold (CT) value (23). The cutoff value of viral shedding was defined as a CT value greater than 35. Negative results were recorded when both N and ORF lab CT values exceeded 35 or were positive. A negative result of each patient was confirmed by repeated testing the next day. Duration of viral shedding was defined as the time from the first qPCR positive diagnosis or the onset of symptoms to the first negative diagnosis (13, 24). To better align with public health decision-making, we divided viral shedding into rapid viral shedding (RVS) and slow viral shedding (SVS) based on the median value of 11.

### 2.3 Statistical analysis

All data were statistically analyzed using R software (version 4.0). For comparison of multiple independent samples, the Kruskal-Wallis test was used. Continuous variables were expressed as mean ± standard deviation (SD) and analyzed using Student’s t-test or one-way ANOVA. Categorical variables were presented as numbers (percentages) and analyzed using Fisher’s exact test. Correlation coefficient was assessed by Spearman correlation. Graphs were generated using the “ggplot2” in R packages. *p* < 0.05 was considered statistically significant.

## 3 Results

### 3.1 Comparison of baseline characteristic between BA.4/5 and BF.7

A total of 542 patients were enrolled in the study, with 118 patients infected with the BA.4/5 variant and the remaining 424 patients infected with the BF.7 variant (Table 1). The mean age of patients in the BA.4/5 group was 28.2 ± 11.2 years, which was significantly lower than the mean age in the BF.7 group (40.200 ± 13.938 years). However, there was no significant difference in gender distribution between the two groups. Duration of viral shedding was shorter in patients infected with BA.4/5 (9.720 ± 2.666 days) compared to those infected with BF.7 (10.750 ± 2.496 days), with the difference being statistically significant (*p* < 0.0001). The proportion of allergic reactions and fever were similar between the two groups, with no statistically significant difference (*p* > 0.05). Although the proportion of asymptomatic infection in BA.4/5 group was slightly higher than that in BF.7 group, this difference was not statistically significant (*p* > 0.05). All participants in BA.4/5 were vaccinated, while a small number in the BF.7 group were unvaccinated. There were no significant differences in the levels of WBC, Hb, PLT, Neu, and Lym between the two groups. The CRP level was lower in BA.4/5 group than that in BF.7 group, but the difference was not statistically significant (*p* > 0.05). In terms of vaccine status, the overall vaccination rate differed significantly between the BA.4/5 and BF.7 groups (*p* < 0.0001). However, there were no significant differences in vaccination rates among the three vaccine types (CoronaVac, BIBP, and Others) (*p* > 0.05) (Table 1). Table 2 further indicates that there were no significant differences in the vaccination rates or the vaccination times for the three vaccine types (CoronaVac, BIBP, and Others) between the SVS and RVS groups (*p* > 0.05).

**Table 1:** Baseline of Omicron patients’ characteristics in the BA.4/5 and BF.7 groups.

Characteristic	Level	Overall	BA.4/5	BF.7	*p*
*n*		542	118	424	
VS (mean (SD))		10.526 (2.567)	9.720 (2.666)	10.750 (2.496)	0.0001
Age (mean (SD))		37.598 (14.255)	28.246 (11.157)	40.200 (13.938)	<0.0001
Sex (%)	Female	231 (42.62)	49 (41.53)	182 (42.92)	0.8677
	Male	311 (57.38)	69 (58.47)	242 (57.08)	
Allergy (%)	No	484 (89.30)	106 (89.83)	378 (89. 15)	0.9658
	Yes	58 (10.70)	12 (10.17)	46 (10.85)	
Fever (%)	No	356 (65.68)	77 (65.25)	279 (65.80)	0.999
	Yes	186 (34.32)	41 (34.75)	145 (34.20)	
Diagnose (%)	Asymptomatic infection	304 (56.09)	76 (64.41)	228 (53.77)	0.0507
	Mild	238 (43.91)	42 (35.59)	196 (46.23)	
Vaccine (%)	No	81 (14.94)	0 (0.00)	81 (19. 10)	<0.0001
	Yes	461 (85.06)	118 (100.00)	343 (80.90)	
WBC (mean (SD))		5.607 (1.940)	5.525 (1.510)	5.629 (2.042)	0.7462
Hb (mean (SD))		144.357 (17.206)	140.830 (18.201)	145.294 (16.862)	0.1141
PLT (mean (SD))		213.737 (56. 183)	225.830 (38.924)	210.525 (59.616)	0.097
Neu (mean (SD))		3.576 (1.810)	3.671 (1.518)	3.551 (1.882)	0.6876
Lym (mean (SD))		1.403 (0.598)	1.258 (0.543)	1.442 (0.608)	0.0615
CRP (mean (SD))		11.494 (14.596)	8.643 (9.549)	12.260 (15.611)	0.1318
Dose_ 1 (%)	CoronaVac	111 (63.07)	19 (67.86)	92 (62.16)	0.6254
	BIBP	61 (34.66)	9 (32.14)	52 (35.14)	
	Others	4 (2.27)	0 (0.00)	4 (2.70)	
Dose_2 (%)	CoronaVac	107 (61.49)	18 (64.29)	89 (60.96)	0.7711
	BIBP	58 (33.33)	8 (28.57)	50 (34.25)	
	Others	9 (5. 17)	2 (7.14)	7 (4.79)	
Dose_3 (%)	Corona Vac	89 (52.35)	17 (62.96)	72 (50.35)	0.407
	BIBP	49 (28.82)	7 (25.93)	42 (29.37)	
	Others	32 (18.82)	3 (11.11)	29 (20.28)	
time21 (mean (SD))		30.287 (29.999)	26.407 (7.958)	31.014 (32.480)	0.4657
time32 (mean (SD))		227.406 (38.893)	225.083 (37.830)	227.895 (39.259)	0.7488
timeIn1 (mean (SD))		493.227 (136.865)	460.929 (129.989)	499.338 (137.696)	0.174

*n*, number of patients; VS, Viral Shedding; WBC, White Blood Cell; Hb: Hemoglobin; PLT, Platelets; Neu, neutrophils; Lym, Lymphocyte cell; CRP, C-reactive protein; Dose_ 1: The type of the first doses of vaccine; Dose_2: The type of the second doses of vaccine; Dose_3: The type of the third doses of vaccine. Corona Vac: The vaccine of Sinovac Biotech CO., LTD. BIBP: The vaccine of Beijing Institute of Biological Products CO., LTD. time21: The time interval between the second and first doses of vaccine. time32: The time interval between the second and third doses of vaccine. timeIn1: The time interval between admission time and first doses of vaccine. The measurement data of the two groups were analyzed by unpaired t test (two-sided). Multi groups’ measurement data was used for ANOVA test. Enumeration data was used for Fisher’s exact test.

**Table 2:** Baseline of Omicron patients’ characteristics in the SVS and RVS groups.

Characteristic	Level	Overall	RVS	SVS	*p*
*n*		542	264	278	
VS (mean (SD))		10.526 (2.567)	8.496 (1.753)	12.453 (1.516)	<0.0001
Age (mean (SD))		37.598 (14.255)	34.659 (13.280)	40.388 (14.607)	<0.0001
Sex (%)	Female	231 (42.62)	101 (38.26)	130 (46.76)	0.0556
	Male	311 (57.38)	163 (61.74)	148 (53.24)	
Allergy (%)	No	484 (89.30)	238 (90.15)	246 (88.49)	0.6264
	Yes	58 (10.70)	238 (90. 15)	32 (11.51)	
Group (%)	BA.4/5	118 (21.77)	85 (32.20)	33 (11.87)	<0.0001
	BF.7	424 (78.23)	179 (67.80)	245 (88. 13)	
Fever (%)	No	356 (65.68)	171 (64.77)	185 (66.55)	0.7306
	Yes	186 (34.32)	93 (35.23)	93 (33.45)	
Diagnose (%)	Asymptomatic infection	304 (56.09)	156 (59.09)	148 (53.24)	0. 1985
	Mild	238 (43.91)	108 (40.91)	130 (46.76)	
Vaccine (%)	NO	81 (14.94)	33 (12.50)	48 (17.27)	0. 1,513
	Yes	461 (85.06)	231 (87.50)	230 (82.73)	
WBC (mean (SD))		5.607 (1.940)	5.692 (1.598)	5.532 (2.202)	0.5405
Hb (mean (SD))		144.357 (17.206)	143.971 (16.465)	144.697 (17.896)	0.7534
PLT (mean (SD))		213.737 (56. 183)	218.829 (57.367)	209.244 (54.967)	0.2033
Neu (mean (SD))		3.576 (1.810)	3.674 (1.553)	3.490 (2.011)	0.4508
Lym (mean (SD))		1.403 (0.598)	1.380 (0.584)	1.423 (0.612)	0.5957
CRP (mean (SD))		11.494 (14.596)	11.528 (14.214)	11.464 (14.991)	0.9743
Dose_ 1 (%)	Corona Vac	111 (63.07)	56 (65.88)	55 (60.44)	0.7377
	BIBP	61 (34.66)	27 (31.76)	34 (37.36)	
	Others	4 (2.27)	2 (2.35)	2 (2.20)	
Dose_2 (%)	Corona Vac	107 (61.49)	53 (62.35)	54 (60.67)	0.1495
	BIBP	58 (33.33)	25 (29.41)	33 (37.08)	
	Others	9 (5. 17)	7 (8.24)	2 (2.25)	
Dose_3 (%)	Corona Vac	89 (52.35)	46 (56. 10)	43 (48.86)	0.5489
	BIBP	49 (28.82)	23 (28.05)	26 (29.55)	
	Others	32 (18.82)	13 (15.85)	19 (21.59)	
time21 (mean (SD))		30.287 (29.999)	28.671 (25.527)	31.775 (33.676)	0.5006
time32 (mean (SD))		227.406 (38.893)	224.638 (38.574)	230.174 (39.294)	0.4051
timeIn1 (mean (SD))		493.227 (136.865)	487.094 (143.866)	498.956 (130.527)	0.5671

RVS, rapid viral shedding. SVS, slow viral shedding.

### 3.2 Comparison between rapid viral shedding (RVS) and slow viral shedding (SVS)

Based on the median of viral shedding, 542 patients were divided into two group: the RVS group (*n* = 264) and the SVS group (*n* = 278) (Table 2). There was a significant difference in viral shedding duration between RVS group and SVS group (*p* < 0.0001). The proportion of males was higher in RVS group compared to SVS group. The incidence of allergic reactions and fever did not differ significantly between the two groups. When comparing the viral shedding duration between BA.4/5 and BF.7 groups, a significant difference was observed (*p* < 0.0001). For the other indicators, including fever, diagnosis, vaccination status, WBC, Hb, PLT, Neu, Lym, CRP, type of vaccine doses and interval time between vaccinations, no statistically significant differences were found (*p* > 0.05).

### 3.3 Correlation between viral shedding and immune indicators

**Figure 1:**
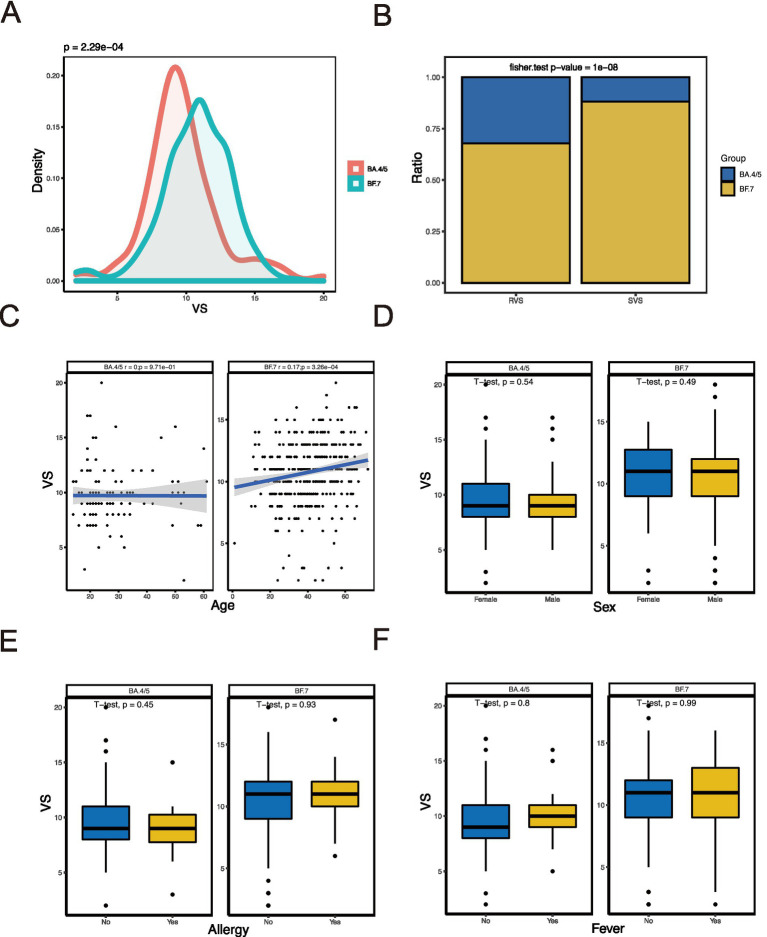
Relationship of VS with baseline characteristics. **(A)** Comparison of VS between patients with BA.4/5 and patients with BF.7. **(B)** Comparison RVS and SVS proportion in BA.4/5 and BF.7 groups. **(C)** Correlation analysis between VS and age. **(D)** Comparison of VS between female patients and male patients. **(E)** Comparison of VS between patients without allergy and patients with allergy. **(F)** Comparison of VS between patients without fever and patients with fever.

**Figure 2:**
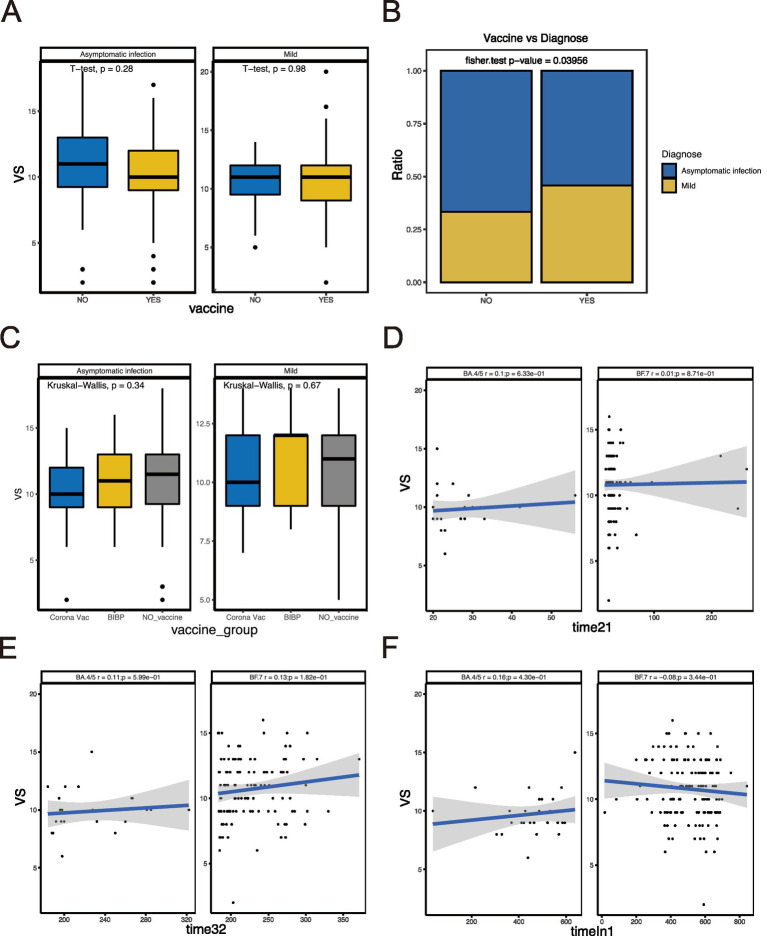
Relationship between VS and vaccination-related indicators. **(A)** Comparison of VS between patients vaccinated and not vaccinated. **(B)** Comparison of the proportion of asymptomatic and mild patients in vaccinated and unvaccinated groups. **(C)** Comparison of VS between different vaccine types. **(D–F)** Correlation analysis between VS and interval time of vaccination.

**Figure 3:**
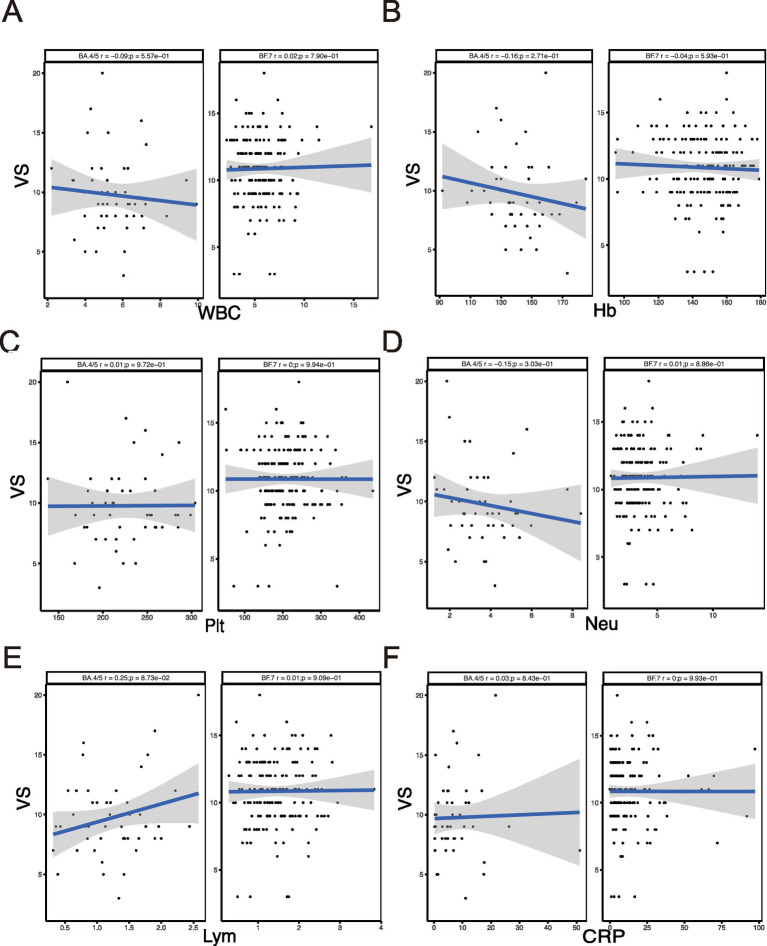
Correlation between VS and immune-related indicators. **(A–F)** Correlation analysis between VS and WBC, Hb, PLT, Neu, Lym, CRP.

We compared the difference between BA.4/5 and BF.7 variants (Figure 1A) and analyzed the correlation between viral shedding and the immune landscape associated with these two variants. In SVS and RVS group, the proportion of patients infected with BF.7 variants was higher than those infected with BA.4/5 variants, which was statistically significant (Figure 1B). Regarding age, there was no correlation between viral shedding and age in BA.4/5 group, whereas a positive correlation was observed in BF.7 group (Figure 1C). No statistically significant difference in viral shedding duration was found between male and female in either the BA.4/5 or BF.7 group (Figure 1D). Moreover, neither allergy nor fever affected VS in either variant group (Figures 1E,F). In addition, whether the patients were asymptomatic or mild symptoms, and whether they were vaccinated or not, had no effect on viral shedding (Figure 2A). A greater proportion of asymptomatic individuals were unvaccinated (Figure 2B). We also compared the duration of viral shedding among asymptomatic patients and mild patients across vaccine types, but found no statistically significant differences between the groups (Figure 2C). Besides, we analyzed the correlation between viral shedding and interval time between vaccinations (time21 and time32) for both variants, finding a positive correlation (Figures 2D,E). Viral shedding was negatively related to timeIn1 in BF.7 group, but positively correlated in BA.4/5 group (Figure 2F). We further examined the relationship between viral shedding and blood routine parameters, as well as CRP levels, in the two variants groups. The relationship between viral shedding and WBC was opposite between the two groups: a negative correlation in the BA.4/5 group and a positive correlation in the BF.7 group (Figure 3A). Viral shedding was negatively correlated with Hb in both BA.4/5 and BF.7 groups (Figure 3B). A positive correlation between viral shedding and PLT was observed in the BA.4/5 group, but no correlation was found in the BF.7 group (Figure 3C). Viral shedding was negatively related to Neu in BA.4/5 group and positively correlated in BF.7 group (Figure 3D). Furthermore, there was a positive relationship between viral shedding and Lym in both the two groups, with a stronger correlation in BA.4/5 group (Figure 3E). Additionally, viral shedding was positively correlated with CRP in BA.4/5 but showed no relevance to BF.7 group (Figure 3F).

## 4 Discussion

Three years have passed since the onset of the COVID-19 pandemic, during which time SARS-CoV-2 has undergone continuous mutation. In our study, we explored the relationship between viral shedding and immune-related indicators in BA.4/5 and BF.7 variants. Our findings demonstrated no significant relationship between viral shedding and immune indicators, including WBC, Hb, PLT, Neu, Lym, CRP and vaccine-related indicators. While viral infections generally prime the body’s immune response, and the duration of viral shedding is closely correlated with the infectivity of the virus (25, 26). Thus, it is necessary to investigate the relationship between viral shedding and immune evasion mechanisms.

Interestingly, our study found no association between viral shedding and factors such as gender, age, allergy status, or the presence of fever. However, Xu et al. reported that males were significantly associated with prolonged SARS-CoV-2 RNA shedding (13), attributing this finding to a higher likelihood of smoking among men. On the contrary, a prospective study suggested that females were more likely to experience extended viral shedding (27), possibly due to a tendency for a stronger Th2-driven immune response in females compared to males (28). Our results differ from both of these studies, which could be due to the variants we studied having a greater capacity for immune evasion, leading to a reduced immune response to the virus. Moreover, the mean age of patients in the RVS group was significantly younger than those in the SVS group, aligning with previous studies (11, 29). This observation may be related to immunosenescence, the age-related decline in immune function, which is more pronounced in older individuals (30). While patients with allergy are more likely to experience immune dysregulation, our study found no correlation between allergy status and viral shedding, which might further suggest the potential for immune escape by the variants. A study showed that higher body temperatures were associated with prolonged viral shedding (18), linking this to the host immune response. However, in our study, the presence of fever did not significantly affect viral shedding. Since fever is a component of the immune response to acute infection, this lack of correlation might be due to the two Omicron variants’ ability to induce a weaker immune response through immune evasion.

Additionally, we analyzed vaccination-related indicators and found that viral shedding is not associated with vaccination status, vaccine types or interval time between vaccinations. The results were consistent with the study of Q.L. Hua et al. (19), which reported that receiving two or three doses of a vaccine did not shorten viral shedding in patients infected with the Omicron variant. Although their study focused on a different Omicron subvariant (BA.2.38), two of the vaccine types they used, BIBP and CoronaVac, were also utilized in our study, yielding similar results. Besides, we also analyzed the interval time between vaccinations, which showed no effect on viral shedding. We deduced that despite the differences in variants, and even though our study focused on more recent variants, this suggests that Omicron has demonstrated immune escape capabilities for an extended period. According to the Centers for Disease Control and Prevention, at least 36 mutations in the spike (S) protein of Omicron are crucial for its infectivity and ability to evade the immune system. A study published in Nature Microbiology also confirmed that Omicron was an immune escape variant (31), further supporting our results.

Moreover, Neu are the first responders in immune cells following viral invasion. Our results showed no significant association between viral shedding and several immune indicators, including WBC, Hb, PLT, Neu, Lym and CRP. This finding contrasts with the result reported by S.J. He et al. (15), who identified that elevated Neu, decreased Hb and decreased Lym as risk factors for prolonged time to viral clearance and as being highly associated with extended viral shedding. This discrepancy suggested that BA.4/5 and BF.7 variants may have a greater capacity for immune escape, resulting in a milder immune response insufficient to reach statistical significance (32). An early study on the characteristics of peripheral lymphocyte subsets in COVID-19 patients from Wuhan also suggested that CD8 T cell and CD4/CD8 ratio are strongly associated with inflammatory status in COVID-19 (33). This indicated a need for further exploration of the relationship between peripheral lymphocyte subsets and viral shedding in the context of the two variants studied here. CRP was significantly related to severity of COVID-19 patients (34). Previous studies have shown that higher CRP levels are observed in patients with prolonged viral shedding (14), likely due to impaired immune function. Our results, however, suggested the opposite, which may be attributed to differences in study populations; their study focused on early COVID-19 patients in Wuhan, whereas our population consisted of patients infected with Omicron variants. In addition, a study examining difference in viral shedding between symptomatic and asymptomatic SARS-CoV-2 infected patients found that asymptomatic individuals exhibited higher WBC levels, lower CRP levels, and shorter viral shedding durations (35). This finding also contrasts with our result. It was possible that the immune escape mechanisms associated with viral mutations allow the virus to reduce immune activity, thereby preventing an overt immune response.

There are certain limitations to our study. We focused only on commonly used laboratory tests and clinical measurements and had limitations in the data currently available from the patients, we were unable to explore immune-related clinical markers, so it will be a focus of our future research efforts. Additionally, to enhance the generalizability of our findings, future studies would benefit from a multi-center approach with a larger and more diverse cohort, encompassing a broader range of demographic characteristics. This would help address potential biases and ensure more representative results. Furthermore, the mechanisms underlying immune escape in patients infected with Omicron BA.4/5 and BF.7 variants should be further investigated.

## 5 Conclusion

Based on the immune-related indicators analyzed in our study, BA.4/5 and BF.7 are likely immune escape variants. Further studies are necessary to explore the immune escape mechanisms of the BA.4/5 and BF.7 variants in greater detail.

## Data Availability

The original contributions presented in the study are included in the article/supplementary material, further inquiries can be directed to the corresponding authors.
